# Twelve Smartphone Applications for Health Management of Older Adults during the COVID-19 Pandemic

**DOI:** 10.3390/ijerph181910235

**Published:** 2021-09-29

**Authors:** Seong Kyu Ha, Hey Sig Lee, Hae Yean Park

**Affiliations:** 1 Department of Occupational Therapy, Jungwon University, Goesan-gun 28023, Korea; haseongkyu@gmail.com; 2 Department of Occupational Therapy, Yonsei University, Wonju 26493, Korea; yozohzzz@gmail.com; 3 Department of Occupational Therapy, College of Software and Digital Healthcare Convergence, Yonsei University , Won ju 26493, Korea

**Keywords:** applications, COVID-19, health management, older adults, OTPF-4

## Abstract

This study investigated smartphone applications that may be helpful in managing the health of the elderly during COVID-19. The application searched the seven areas of health management, newly classified in OTPF 4th edition with keywords in the Google Play Store. As a result, two applications meeting the selection criteria were selected for each area. The selected applications are social and emotional health promotion and maintenance: *Wysa & MindDoc*, symptom and condition management: *Ada & Diseases Dictionary*, communication with the health care system: *Telehealth & Blood Pressure Diary*, medication management: *Medisafe & MyTherapy*, physical activity: *FitOn & Samsung Health*, nutrition management: *Lifesum & Health and Nutrition Guide.* Through the analyzed applications, twelve applications with the potential to improve the health management and quality of life in older adults during social distancing or self-isolation due to COVID-19 were identified.

## 1. Introduction

The first case of coronavirus disease (COVID-19) was recorded in Wuhan, China, in December 2019. Since then, there have been approximately 71 million confirmed cases and 1.6 million deaths worldwide [1]. The WHO officially declared COVID-19 a pandemic on 12 March 2020, making it the third pandemic following the 1968 Hongkong flu and the 2009 novel influenza A (H1N1). The symptoms of COVID-19 include fever, dry cough, and fatigue and this disease is currently known to be transmitted through droplets generated during coughing or sneezing, or through touching the eyes, nose, or mouth after coming in contact with a COVID-19-contaminated surface [2].

Personal and social restrictions, which limit outdoor activities and facility use, have been imposed for preventing and managing COVID-19. However, these restrictions also endanger the health of the global population, especially older adults. They require special attention and care as they are more susceptible to COVID-19 due to their old age and high likelihood of having comorbidities [3]. In the United States, 8 out of 10 deaths from COVID-19 occurred in older adults aged ≥65 years, confirming that they are at high risk of this disease [4]. Older adults need to consistently participate in physical activities and sustain healthy habits to maintain and manage their health, especially during this pandemic. Maintaining psychological health is also important since quarantining or spending most of the day at home during the pandemic can be highly distressing and cause individuals to experience strong emotions due to anxiety [5]. Therefore, governments must pay close attention and take prompt actions to promote health management in older adults during the pandemic.

Mobile healthcare is becoming increasingly popular as a way to promote health management in older adults during the pandemic [6]. It utilizes smartphones to increase awareness about various methods of health management by providing health-related education, self-diagnostic tests, and information about treatments. Consistent advances in information and communication technologies (ICTs) have led to their increased utilization for mobile healthcare among older adults [7]. Mobile healthcare has advanced from providing health services through phone calls with a healthcare expert to using applications. Mobile applications are more cost-effective than phone calls as they can support an unlimited number of users. They can be used anywhere and anytime, and they automatically analyze health data, save the analysis results, and communicate these results to healthcare experts. A growing number of applications are being utilized in this field. For instance, one application utilizes an older adult-friendly interface through which the older people can communicate with their families and caregivers in real time [8]. Another application records blood pressure, heart rate, and weight, and receives feedback from healthcare professionals [9]. Despite the potential benefits of these applications for older adults in terms of health management, few studies have analyzed these applications in depth.

In order to take a systematic approach to analyzing applications for health management, there are limitations to classification methods such as Google Store’s classification method and filter system. Therefore, the author intends to utilize the latest version of the Occupational Therapy Practice Framework (OTPF) 4th edition as a tool for systematic application analysis. OTPF accounts for a large proportion of research in the field of occupational therapy. In a number of studies, including studies for occupation in mild Traumatic Brain Injury patients, studies for Instrumental Activities of Daily Living (IADL) in Parkinson patients, development of leisure participation evaluation tools for the elderly, and studies for activities and health of the elderly outside the home, OTPF can be seen as an important basis [10,11,12,13]. The Occupational Therapy Practice Framework 4th edition (OTPF-4) includes health management as one of the nine major occupational domains that is independent from all the other domains [14]. This suggests that health management is recognized to be as important as other domains, such as the activities of daily living and instrumental activities of daily living. Furthermore, studies on healthy, active, and successful aging have suggested that health management is an important aspect of aging, especially for older adults [15]. Health management refers to activities aimed toward improving or maintaining one’s health to support participation in other tasks. It is divided into the following seven subdomains: (1) social and emotional health promotion and maintenance, (2) symptoms and condition management, (3) communication with the health care system, (4) medication management, (5) physical activity, (6) nutrition management, and (7) personal care device management. With health management now being considered as an independent domain in the OTPF-4 and the COVID-19 pandemic affecting daily routine substantially, health management in older adults is becoming increasingly important, necessitating an in-depth analysis of health management applications for older people.

In this study, the authors conducted an in-depth analysis of smartphone applications for health management in older adults, based on the subdomains of health management in the OTPF-4. This study provides basic data about existing healthcare applications for older adults that may be used in future application development.

## 2. Methods

### Procedure

The authors examined smartphone applications for health management in older adults which were published on the Google Play Store, 1 December 2020. Applications were chosen based on the inclusion criteria. Applications were finalized after consulting geriatric specialists. Before searching for applications, it was decided that they would be divided into the aforementioned seven subdomains of the healthcare management domain in the OTPF-4.

**Figure 1:**
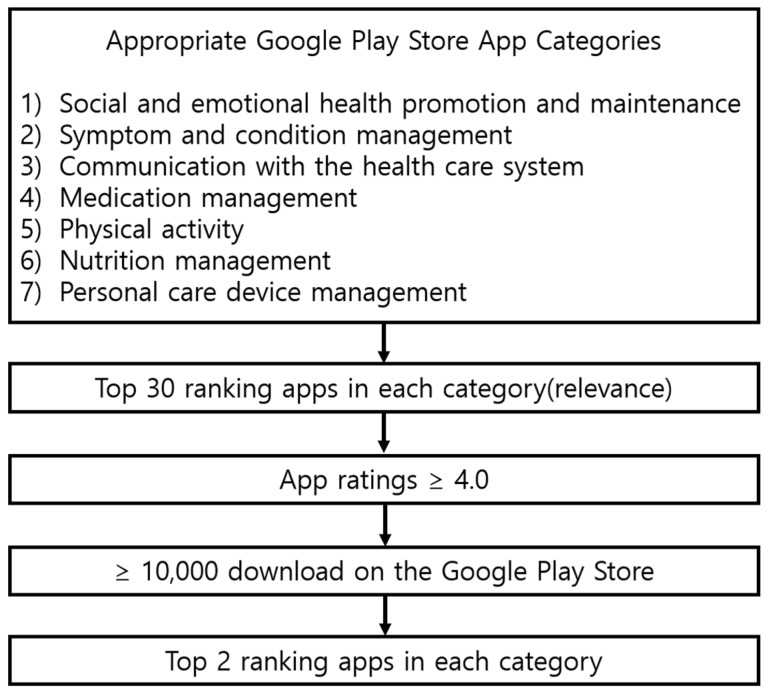
Inclusion and exclusion criteria for 12 smartphone applications for older adults to use during the COVID-19 pandemic.

Applications were selected through the following process. The first round of screening was done by selecting the top 30 results that appeared after typing each of the seven domains in the search bar in the Google Play Store. The second round of screening included selecting only those applications with ratings ≥4 out of 5. In the third round of screening, those applications that were downloaded ≥10,000 times were selected. Classifying applications into subdomains was finalized only after mutual agreement between researchers (Figure 1). The selected application was tested by the authors to confirm that the function described by the developer was actually implemented.

Two applications were chosen for each subdomain. The names, price options, functions, ratings, number of downloads, and reviews of these applications were analyzed. In order to reduce the risk of subjective bias of the researcher, only reviews of real users were used. The review was selected based on the researcher’s discussion and judged to best express the usability of the application. All details on the selected applications were based on the information available on 1 December 2020.

## 3. Results

Twelve applications were finalized after applying the inclusion criteria. Of the seven subdomains of health management in the OTPF-4, personal care device management overlaps with the remaining six subdomains and can be included in applications in the other subdomains. In this study, personal care device management was included as part of the remaining six domains. Table 1 shows the names, prices, and functions of the applications in the six subdomains. Users’ reviews were examined to understand their experience of using the application. The data used publicly available data that does not violate data protection and privacy. ***Samsung Health*** had the highest number of reviews (1,114,000 reviews). The app with the highest overall average of user-provided star ratings was ***Fiton*** with 4.8 stars (Table 2).

## 4. Discussion

Studies have shown the negative effects of the extended COVID-19 lockdown on the health of older adults [16,17]. They have also examined the effectiveness of smartphone applications as an online method of health management for older people [18,19,20]. The addition of the health management domain in the OTPF-4 demonstrates its importance and indicates that people invest a considerable amount of time and effort in it. In this study, mobile applications with high relevance and popularity in this domain were searched and analyzed. Applications for each subdomain have been discussed ahead in detail. Currently, the applications registered in the Google Store do not have individual filtering to check only the applications applicable to the elderly, so it is difficult to find an appropriate application that can be applied to the elderly. Therefore, based on the results of this study, we would like to propose an application that can be helpful in managing the health of the elderly.

### 4.1. Social and Emotional Health Promotion and Maintenance

Social distancing plays a major role in controlling the spread of COVID-19. However, over a long period, it can have negative effects on one’s psychological health [21]. Of the final two applications that meet the selection criteria out of 50, ***Wysa*** is a simple chat bot that allows users to clear their minds and consequently safeguard their psychological and social well-beings. Users can communicate with the bot anonymously and receive the help they need without going to a hospital. However, the help may not be as effective as consulting an expert in real life. ***MindDoc*** allows users to record their emotions, facilitates them in understanding how they feel, and provides visual and auditory data that promote psychological health. ***MindDoc***’s self-diagnostic checklist can be filled out without the help of an expert. Its mindfulness exercises can provide some relief when one is experiencing high levels of psychological stress. A study has reported that mindfulness exercises promote one’s well-being and provides relief in stressful situations, such as the COVID-19 pandemic [22].

Levels of depression, anxiety, and suicidal ideation are increasing among older adults since the COVID-19 outbreak [23]. Older people experience difficulty describing or expressing their emotions [24]. These applications may allow them to reflect on, describe and express their emotions to convert negative emotions to positive ones. Providing the elderly with visual or auditory resources through an easy-to-use application can positively affect their psychological health.

### 4.2. Symptom and Condition Management

COVID-19 has been placing substantial burdens on healthcare professionals [25] as well as on older adults. It has become more difficult for older people to visit a hospital even to check for underlying symptoms of COVID-19 due to its high transmission rate. Of the final two applications that meet the selection criteria out of 250, ***Ada*** is a simple guide that identifies the causes of different health problems and provides medical advice. It has been especially useful during the COVID-19 pandemic. ***Ada*** asks yes-or-no questions to identify where the user is experiencing pain and allows the user to share this information with their family. If, after self-screening, a user suspects that they have a certain disease, they may visit a doctor. By gaining knowledge about different diseases through the ***Diseases Dictionary*** app, users can prevent drug misuse, understand their conditions, and take feasible measures against diseases. The application also prevents users from missing important treatments due to unverified information such as that pertaining to home remedies. However, it must be clearly communicated to the users that the purpose of these applications is to allow older adults to find information about health management and disease prevention and that any diagnosis and treatment must be conducted by a doctor.

### 4.3. Communication with the Health Care System

While virtual health management has gained more attention since the COVID-19 outbreak [26], Telehealth has been popular as an effective treatment method since before this pandemic. It allows users to communicate with healthcare professionals face-to-face anywhere, at any time, and easily check the calendar and connect via video with a single touch. There is an advantage of distance and time by easily connecting health professionals to people who are distant and in isolation, or who have difficulty in taking time to visit themselves.

Blood pressure management is important for older adults. Since hypertension can significantly aggravate cardiovascular diseases, regularly managing blood pressure is important [27]. Of the final two applications that meet the selection criteria out of 250, The application ***Blood Pressure Diary*** allows users to easily record their blood pressure on a daily basis and provides a calendar view of these records. Users can also send their records to their doctors through the application.

### 4.4. Medication Management

Of the final two applications that meet the selection criteria out of 249, ***Medisafe*** reminds caregivers to help older adults take medications according to a schedule. It also reminds older adults to take medications themselves when caregivers cannot visit them due to social distancing or quarantining during the pandemic. The application also provides information about the side effects, including addiction, and allergic reactions, associated with various kinds of medications taken by older people. Another application, ***Mytherapy***, not only reminds users to take medications but also documents blood pressure and the number of steps taken during the day. All this information can be shared with healthcare professionals and families.

### 4.5. Physical Activity

Health promotion and maintenance is important, especially for older adults [28]. However, long-term social distancing during the pandemic has reduced the level of physical activities among older people [29]. Of the final two applications that meet the selection criteria out of 250, ***FitOn*** is a fitness app providing video tutorials on exercises that older adults can follow at home to compensate for their reduced physical activities. Users can choose different workouts based on intensity, time, and purpose.

***Samsung Health*** was the most commonly used of all the applications examined in this study. It allows for the integrated management of physical functions. The application visualizes data about physical activities, sleep, pulse, and calories using graphs, and recommends exercise programs based on the options chosen by the user. It can also be synced to a Samsung watch for additional features.

### 4.6. Nutrition Management

Obesity is associated with a reduced expiratory reserve volume, functional ability, and pulmonary compliance. Breathing is difficult for bedridden patients with abdominal obesity as these patients have reduced diaphragm movements, and subsequently, poor pulmonary functions [30]. Increased levels of inflammatory cytokines associated with obesity can increase the risk of COVID-19 [30]. The prevalence rates of adult obesity and severe obesity in the United States have increased in the past two decades and are currently 41.9% and 9.2%, respectively [31]. These rates can further increase; long-term social distancing and quarantining during the COVID-19 pandemic have been reported to negatively affect psychological functioning and promote obesity [32].

Patients without COVID-19, patients with mild symptoms of COVID-19, and patients who have recovered from COVID-19 must have a balanced diet [33]. Individuals, especially older adults with reduced physical activity, must follow the principles of healthy eating and detailed recommendations regarding the intake of sugar, salt, refined carbohydrates, cooking oil, saturated fat, trans-unsaturated fatty acids, total dietary fiber, and processed or canned foods [33,34,35]. Of the final two applications that meet the selection criteria out of 50, The application ***Lifesum*** displays the nutritional content of different foods, as well as information about calories and nutritional content through its barcode scanning feature. Such information allows users to intuitively understand which nutrients they are lacking and design a balanced diet accordingly. ***Health and Nutrition Guide*** provides information about foods that can cause allergies or aggravate a condition. It suggests foods that can relieve different symptoms with images and provides information about foods and vegetarian diets that can help older adults manage their nutritional status.

### 4.7. Limitations

Among the application platforms, Apple store was excluded because it did not provide information on the application selection criteria in this study and therefore may have excluded some apps used by many users of smartphones. The relevance to the OTPF domain, a filter option available at the Google Play Store, was used to filter applications in this study. It was not possible to determine the exact algorithm by which relevance was determined. Furthermore, the authors could not filter the applications by users’ age, even though our study was examining smartphone applications for older adults. In the reviews of the analyzed applications, there were many contents that were easy to use and easy to operate, however their usability can still vary depending on older people’s cognitive and physical level of functioning. Additionally, this study has little implication for older adults who do not own a smartphone.

## 5. Conclusions

Health management is becoming increasingly important for older adults due to the extended period of social distancing and quarantine during the COVID-19 pandemic. At the same time, maintaining health-promoting behaviors by oneself, or receiving healthcare services is becoming difficult. Since old age and comorbidities can increase the incidence and severity of COVID-19, actively managing the health of older adults may be one of the ways to prevent the spread of COVID-19. With the extended period of social distancing and quarantine, it is difficult to maintain one’s own efforts for health management or the health care services that have been received face-to-face in the community. The authors examined smartphone health management applications that may provide a solution to this problem. Twelve applications were analyzed that met the inclusion criteria and were all found to be useful for health management in older adults. Managing health will not necessarily prevent the “spread” of COVID-19 but may impact the psychological and social wellbeing of elderly individuals impacted by COVID-19, and by all older adults interested in improving their quality of life and aging in place. While the number of older smartphone users is growing [36], there is still a large population of older adults who cannot use smartphones due to financial burdens, complicated methods of use, poor vision, or cognitive impairment. Thus, new approaches for these users can be explored by future studies. In addition, professionals, including occupational therapists, can recommend these applications to caregivers and older adult patients for easy data tracking and sharing.

## Figures and Tables

**Table 1:** Costs and functions of 12 smartphone applications for older adults to use daily while in isolation during the COVID-19 pandemic.

Domain	App Name	Option	Manufacturer	Function
Social and emotional health promotion and maintenance	Wysa	Free to download. Therapist access (Weekly) US$29.99, Therapist access (Monthly) US$79.99, Therapist access (Quarterly) US$144.99, Wysa Premium (Annual) US$99.99, 1:1 Life Coaching Sessions US$47.99, Wysa Premium (Monthly) US$11.99	commercial company, healthcare institution	Users can talk to AI penguins to better cope with psychological disorders, whether it is to manage stress or seek to improve mental health, treatment-based technology and dialogue create an appealing and calm therapeutic chat app.
	MindDoc: Mood Tracker for Depression & Anxiety	Free to download. Per item US$6.49–US$84.99	commercial company	MindDoc is a leading monitoring and self-management application for mental health disorders like depression, anxiety, eating disorders and stress.
Symptom and condition management	Ada-your health companion	All free	commercial company, healthcare institution	Ada helps users and their families in checking symptoms and discovering what might be causing them. It doesn’t require an appointment and can be used at any time. Its symptom checker can help users in assessing if their symptoms require them to see a doctor.
	Diseases Dictionary	All free	commercial company	Detailed description of major medical conditions and diseases: definition; symptoms; causes; risk factors; complications; preparing for your appointment; tests and diagnosis; treatments and drugs; lifestyle and home remedies
Communication with the health care system	Telehealth by SimplePractice	All free	commercial company	Face-to-face video therapy with a specialist. Connect anytime, anywhere. From calendar to video call in one tap. Simple for you and your client. Get started with one link.
	Blood Pressure Diary	All free	commercial company	It helps in evaluating blood pressure on a daily basis, provides information on blood pressure management and displays blood pressure in a calendar which can be communicated to a doctor.
Medication management	Medisafe	Free to download. Per item US$2.99–US$39.99	commercial company, healthcare institution	It is a medication time reminder and medication management application which helps in taking medications safely and on time. Users can also use this application to help their family take medicine.
	MyTherapy	All free	commercial company, healthcare institution	Body weight, blood pressure, blood sugar levels, etc. can be measured and recorded through this application, enabling systematic management of most diseases (diabetes, rheumatoid arthritis, depression and high blood pressure among others).
Physical activity	FitOn-Free Fitness Workouts & Personalized Plans	Free to download. Per item US$14.99–US$119.99	commercial company	It assists in reaching user goals with personalized workout plans. Goals can include weight loss, building muscles, increasing cardio endurance or reducing stress.
	Samsung Health	All free	commercial company	It checks and records overall physical activity, such as walking, running, biking, and indoor and outdoor sports.
Nutrition management	Lifesum - Diet Plan, Macro Calculator & Food Diary	Free to download. Per item US$3.75–US$52.56	commercial company	It helps in setting diet plans and giving diet tips for any goal. It includes calorie counter and food tracker with barcode scanner for easy logging. Its macro calculator keeps tabs on daily macros, nutrition and calories. Its food planner provides recipes for any plan.
	Health and Nutrition Guide & Fitness Calculators	Free to download. US$2.00 per item	commercial company	It provides information on foods for users at risk of allergies or diseases. It includes image information about foods that may be helpful for each symptom and information on vegetarian food in general.

**Table 2:** Ratings and user reviews of 12 smartphone applications for older adults to use daily while in isolation during the COVID-19 pandemic.

Domain	App Name	Ratings	User Comments
Social and emotional health promotion and maintenance	Wysa	4.7 stars; 66.4 K ratings	The app does a good job of clearing your mind and making you rationalize your thoughts. It’s very soothing and the AI is quite intelligent. I’m impressed with how well it communicates and responds to hard dialogue. Obviously it is not perfect and there is always room for improvement, but it’s still impressive.
	MindDoc: Mood Tracker for Depression & Anxiety	4.6 stars; 34.8 ratings	The best mindfulness/meditation/mental health app I’ve used, and I’ve used a lot. I usually don’t pay for app subscriptions, but this one is worth it. It incorporates more specifically targeted practices than most apps, and keeping track of your mood with very thoughtful questions is really helpful.
Symptom and condition management	Ada-your health companion	4.7 stars; 287 K ratings	Ada is an intelligent app. It is a sure guide to the identification of possible causes of people’s health issues. It is a veritable instrument of medical advice especially during the COVID-19 pandemic. It is a near-perfect effort which all the users of android phones must identify with to help themselves and their loved ones get useful advice concerning their health.
	Diseases Dictionary	4.4 stars; 28.2 K ratings	We should consult a doctor when we suspect a disease and this app is no alternative. But it helps in memorizing the symptoms of few common diseases and is a knowledge provider. We come across many new diseases every day and this helps in knowing about them and the steps we must take to prevent them. Thanks.
Communication with the health care system	Telehealth by SimplePractice	4.4 stars; 5 K ratings	Telehealth has allowed me the opportunity to have amazing therapy sessions during this time of pandemic. In fact even when we can meet face-to-face I think I might still use Telehealth because of how well it works with my schedule. It saves me time and money that I would usually spend driving to mine.
	Blood Pressure Diary	4.7 stars; 35.2 K ratings	Brilliant! It records your BP, gives you history and tells you which range your BP is in – pre-hypertension, stage 1/2 or normal, if you’re fortunate! Plus you can email your records straight to your doctor. I really like this app and it is easy to use too!
Medication management	Medisafe	4.6 stars; 208 K ratings	I have used this app for many years. I have had several TBI’s and this app has been there for me to remind me when to take my meds. My docs love it ‘cause I can show them or ramble off my list of meds for them. Some have shared this app even with their patients. It is relatively easy to use & update. It also has a friend reminder so someone else can remind you too. I love the tracker system in it so I am able to look back & see what meds I have missed. I’m even able to track docs & refills. Best app ever!
	MyTherapy	4.7 stars; 86.5 K ratings	It does everything I need it to while managing to remain unobtrusive. And when I say everything, I mean everything. Not only does it track my medications, blood pressure readings and symptoms, but I can even export them into a clean PDF for my doctor. It is an incredibly convenient way to manage my health when I have to coordinate care between multiple providers.
Physical activity	FitOn-Free Fitness Workouts & Personalized Plans	4.8 stars; 26.3 K ratings	Being at home and never knowing when my Gym will be open, this app rocks! Challenges weekly, daily and you can customize your workouts according to length, type and level of difficulty. There are so many Coaches to choose from as well. You can even join groups for support and in home workouts. I have tried many a fitness App over the past years, but this one by far exceeded my expectations. You can also upgrade and join for very low$ or just continue with basic options. Either way FitOn won’t disappoint.
	Samsung Health	4.0 stars; 1114 K ratings	This app is user friendly. You can customize it to your needs. It helps you monitor stress, sleep, heart rate and blood pressure. Follow the instructions and you will have a somewhat accurate result to how this app is designed for your use. I enjoy this app as much as an everyday user. It helps me monitor my calorie intake (not that I am worried, it gives me an idea of what I am eating and how much I should be eating more of).
Nutrition management	Lifesum-Diet Plan, Macro Calculator & Food Diary	4.5 stars; 257 K ratings	It is very user friendly and does so much more than simply logging food. It is well laid out. Gives you insights on your eating habits, gentle encouragement on where changes are needed and the nutritional info for foods is amongst the best you will find. The food plans are great too if like me you don’t like being tied to recipes!
	Health and Nutrition Guide & Fitness Calculators	4.5 stars; 1.7 K ratings	It is very informative about what foods help on what health issues. Give good insight on pros and cons of many fruits and vegetables. I like it.

## Data Availability

The data used in this analysis from the google play store and are available on its web page http://play.google.com/store/apps (accessed on 1 December 2020).
